# Dietary intakes and biomarker patterns of folate, vitamin B_6_, and vitamin B_12_ can be associated with cognitive impairment by hypermethylation of redox-related genes NUDT15 and TXNRD1

**DOI:** 10.1186/s13148-019-0741-y

**Published:** 2019-10-11

**Authors:** Yu An, Lingli Feng, Xiaona Zhang, Ying Wang, Yushan Wang, Lingwei Tao, Zhongsheng Qin, Rong Xiao

**Affiliations:** 1 School of Public Health, Capital Medical University, No.10 Xitoutiao, You An Men Wai, Beijing, 100069 China; 2 Peking University First Hospital, Beijing, China; 3 Jincheng People’s Hospital, Jincheng, China

**Keywords:** Folate, Vitamin B_12_, DNA methylation, Oxidative stress, Cognitive impairment

## Abstract

**Background:**

B vitamins in the one-carbon metabolism pathway (folate, vitamin B_6_, and vitamin B_12_) have been implicated in DNA methylation, and their deficiency may contribute to cognitive decline through increased homocysteine (Hcy) levels and subsequent oxidative damage. The aim of this study was to investigate whether B vitamin deficiency and increased Hcy could interact with DNA methylation of oxidative-related genes and exacerbate cognitive impairment.

**Methods:**

Participants were selected from a large cohort study entitled the Effects and Mechanism Investigation of Cholesterol and Oxysterol on Alzheimer’s disease (EMCOA) study. We included 2533 participants who completed a selection of comprehensive cognitive tests and a semiquantitative food frequency questionnaire (FFQ) and were followed for an average of 2.3 years. The longitudinal effects of B vitamin intake on cognitive decline were examined using linear mixed-effect models. Seven mild cognitive impairment (MCI) patients, in the predementia stage of Alzheimer’s disease (AD), and fivev healthy controls were selected for the discovery of genome-wide differentially methylated CpG sites. Candidate oxidative stress-related genes significantly correlated with serum levels of B vitamins were selected for validation in 102 MCI patients and 68 controls. The correlations between DNA methylation levels and serum concentrations of B vitamins and oxidative biomarkers were analyzed with Spearman’s correlation. The interactive effects of DNA methylation and B vitamins on cognitive performance were further evaluated by multiple linear regression.

**Results:**

In the prospective analysis, inadequate dietary intake of vitamin B_12_ was significantly associated with accelerated cognitive decline, whereas adequate folate, vitamin B_6_, and vitamin B_12_ intakes were significantly associated with better cognitive reserve. In the case-control analysis, the DNA methylation levels of NUDT15 and TXNRD1 were examined, and significantly hypermethylated sites were identified in MCI patients. Significant correlations of hypermethylated sites with serum levels of folate, homocysteine (Hcy), and oxidative biomarkers were observed, and interactive effects of B vitamins and hypermethylated sites were significantly associated with cognitive performance.

**Conclusion:**

Adequate dietary folate at baseline predicted a better cognitive reserve, while decreased serum levels of B vitamins may contribute to cognitive impairment by affecting methylation levels of specific redox-related genes.

**Trial registration:**

EMCOA, ChiCTR-OOC-17011882, Registered 5th, July 2017-Retrospectively registered, http://www.medresman.org/uc/project/projectedit.aspx?proj=2610

**Graphical Abstract:**

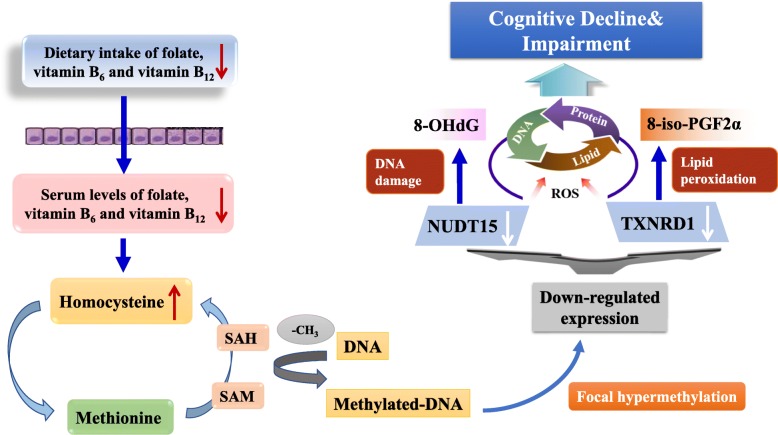

## Background

Alzheimer’s disease (AD) is a chronic and progressive disorder causing deterioration of cognitive function beyond the normal aging process among older people worldwide, ranging in severity from predementia stages such as mild cognitive impairment (MCI) to clinical stages of dementia [1]. As a leading chronic disease contributing to disability and dependence, AD is characterized by progressive cognitive decline and growing functional impairment, beginning with mild difficulties with instrumental activities of daily living (ADL), such as using a telephone and managing medication, and ending with the loss of basic ADL, such as bathing, eating, and dressing [2].

MCI is considered to be a transitional stage between healthy aging and dementia, characterized by cognitive deficits in several domains greater than expected for an individual’s age but slight or no impairment in instrumental ADL [3]. Dementia refers to severe brain disorders associated with largely generalized cognitive dysfunction, behavioral disturbances, loss of basic ADL, disability and dependency associated with personal, social, and economic burden [4]. Such concomitant cognitive and functional difficulties increase dependence to negatively affect quality of life (QOL), which is a multidimensional construct integrating cognitive function, physical function, social interactions, mental well-being, and mood [5].

The World Alzheimer Report 2018 has estimated that 46.8 million people were suffering from dementia globally in 2015, and the number is predicted to triple by 2050 [6]. Consequently, AD presents continuous social and economic challenges with an ever-increasing aging population. It has been acknowledged that the neurodegenerative process during AD is inevitably irreversible, but early intervention in MCI, including decreasing the risk factors, is promising [7]. As a result, the exploration of strategies to prevent or delay the onset of AD, including identification of risk factors of MCI, has become a major priority of global public health.

There is no doubt that the development of MCI/AD in later life is affected by a series of modifiable risk factors associated with lifestyle and nutritional status [8], such as smoking, education, reading, and dietary nutrient patterns [9]. Potential underlying mechanisms have been proposed, including antioxidant defense, anti-inflammatory effects, decreased vascular burden, and altered DNA methylation in the central nervous system [10]. In particular, a copious amount of epidemiological evidence has suggested that suboptimal status of B vitamins contributes to cognitive dysfunction in the elderly. The deficiency of B vitamins has been commonly reported in the elderly, which may be caused by inadequate intake, drug-nutrient interactions, and increased requirements due to the negative effects of aging on the absorption, transport, and metabolism of B vitamins [11]. Epidemiological findings of the associations of B vitamin intakes with domain-specific cognitive decline as well as risk of MCI/AD are incongruent. Kim et al. [12] investigated the relationship between intakes of B vitamins and cognitive function in 100 subjects with MCI, 100 with AD, and 121 normal subjects. The findings of revealed that total B vitamin intake was associated with better global and domain-specific cognitive function in the AD and MCI groups. However, a large prospective study that enrolled a total of 3718 residents aged 65 years and older found that higher intake of folate may be harmful in relation to cognitive decline [13], which may be attributed **to** much higher intake of total folate (from food and supplements) than the dietary reference intakes (DRIs) after the implementation of a folic acid fortification program in the USA and residual confounding caused by factors associated with higher folate intake and slower cognitive decline. Moreover, there has been evidence in randomized controlled trials (RCT) to show different effects of B vitamin supplementation on cognitive function in the elderly, and a recent meta-analysis concluded that B vitamin supplementation had no effect on cognition [14].

Although no clear mechanisms have been well established, several biologically plausible mechanisms have been proposed to explain the role of one-carbon metabolism-related B vitamins, including folate, vitamin B_12_, and vitamin B_6_, in cognitive dysfunction [15]. Deficiencies in any of these B vitamins might raise the blood concentration of homocysteine (Hcy) by perturbing one-carbon metabolism and leading to low enzymatic activities for the remethylation or trans-sulfuration of homocysteine [16]. Methylation is a key mechanism by which the body deals with toxins, stress, and infections. The results of ineffective methylation reactions may contribute to numerous diseases, including neurological disorders. B vitamins are essential in the synthesis of *S*-adenosyl-methionine (SAM), which is required for methylation of DNA [17]. It has been well established that vitamin B_6_, folate, and vitamin B_12_ in the diet can reduce serum Hcy level and promote its remethylation to methionine. Consequently, lower intake of these B vitamins and elevated serum Hcy level are linked to altered DNA methylation patterns, which has been observed in AD patients [18]. As a neurotoxin, an increased level of Hcy has also been shown to affect redox signaling pathways in neurons by generating reactive oxygen species (ROS) and decreasing endogenous antioxidants [19]. It is therefore of interest to see whether DNA methylation patterns in redox-related genes could modulate cognitive impairment conferred by B vitamin deficiency and hyperhomocysteinemia.

The present study was designed to investigate the effects of dietary intakes of these one-carbon metabolism-related B vitamins on global and domain-specific cognitive decline in a large Chinese cohort and then explore the possible role of DNA methylation of genes in oxidative stress pathways as a mediator in a case-control design. The results of this study will provide a theoretical basis and scientific data for uncovering the potential interactions between B vitamin nutritional status and the genetic background of cognition.

## Materials and Methods

### Participants

Participants between the ages of 50 and 70 years were selected from a large-scale community-based study entitled the Effects and Mechanism investigation of Cholesterol and Oxysterol on Alzheimer’s disease (EMCOA) study, an ongoing multicenter epidemiological survey funded by the State Key Program of National Natural Science Foundation of China [20]. This study was registered at the Chinese Clinical Trial Registry as ChiCTR-OOC-17011882. The medical Ethics Committee of Capital Medical University (No. 2013SY35) approved the study protocol, and written informed consent was obtained from all subjects. The baseline examination took place between January 2014 and December 2015, and follow-up examinations took place approximately every 2 years. Face-to-face interviews were performed at each examination, with the collection of sociodemographic information (e.g., age, sex, and education years), medical history of chronic diseases (e.g., hypertension, diabetes, and heart disease), lifestyle (e.g., smoking and drinking), and a broad range of neuropsychological tests and dietary surveys. Fasting venous blood was collected during all the interviews following standardized protocols for storage of blood samples. A survey team consisted of clinical neuropsychologists, and research surveyors were trained in the details of the measurements and questionnaires before starting the examination. We adopted outreach efforts through oral and written advertisements, flyers, word of mouth, and educational presentations provided in the community. The exclusion criteria for the original study included suffering from severe diseases or conditions known to affect cognitive function (e.g., depression, malignant tumors, a history of traumatic brain injury, cerebral infarction or cerebrovascular disease, long-term frequent intake of drugs and medications, or dietary supplements to improve cognitive function). Consequently, the longitudinal association of dietary B vitamins with cognitive decline was investigated among the 2533 participants, who were followed for an average of 2.3 years after the dietary assessment at baseline.

**Fig. 1:**
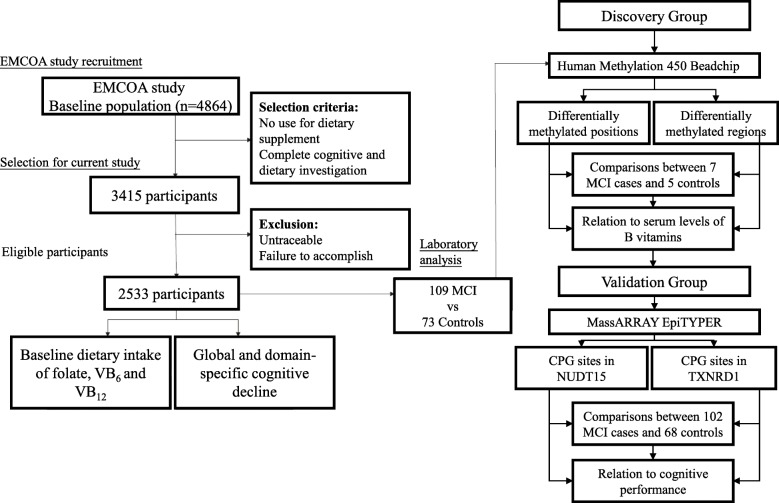
Study flow chart

Next, a subgroup of 109 newly diagnosed MCI patients and 73 controls were selected for DNA methylation and biochemical analyses among participants who were the first to complete the follow-up in 2016 in one center. To further exclude potential confounders and obtain a relatively homogenous study population, the predefined selection criteria included the following: no reported changes in the use of dietary supplements containing B vitamins during the study, no heavy alcohol use at baseline or during follow-up, and no gastrointestinal diseases. Figure 1 describes the procedure for the current study.

### Cognitive tests

The cognitive tests were administered and scored according to a standard procedure by nurses or researchers who had attended unified training several times before. This comprehensive neuropsychological battery evaluated global and multiple cognitive domains, and the time required was approximately 40 min. The Mini-Mental State Examination (MMSE) and Montreal Cognitive Assessment (MoCA) were used for global cognitive evaluation [21]. The Symbol Digit Modalities Test (SDMT) [22] was used to assess processing speed. The Auditory Verbal Learning Test (AVLT) [23], including five trials of the recall of a 12-word list, measured immediate recall (AVLT-IR), short recall (AVLT-SR), and long recall (AVLT-LR) of memory. The Logical Memory Test (LMT) [24] and the digit span forwards (DSF) and digit span backwards (DSB) [25] of the Wechsler Memory Scale-Revised, Chinese version (WMS-RC) were used to measure attention.

### Diagnosis of MCI

MCI patients were diagnosed through a two-stage procedure. First, the cognitive function of subjects was assessed by MoCA, which was a 30-point global cognitive screening instrument used for screening MCI patients [26]. The cut-off points used for MCI screening applied to the elderly Chinese population were as follows: 13/14 for illiterate individuals, 19/20 for individuals with 1 to 6 years of education, and 24/25 for individuals with seven or more years of education [27]. Second, participants suspected of having MCI based on their MoCA performance were secondarily examined by neurologists to establish a clinical diagnosis.

### Dietary assessment

Dietary information was collected from a 33-item interviewer-administered Food Frequency Questionnaire (FFQ). The participants were requested to state the frequency (per year, month, week, or day) and amount (in grams, bowls, etc.) of food intake for each food item during the 1 year before the interview. For each food reported, food models and an album with over 50 photos of the most common dietary products were used as helpful tools to determine the amounts of food portions. To calculate dietary intakes of the relevant B vitamins, the B vitamin composition of each food was multiplied by the frequency of consumption and then summed over all food items. B vitamin composition was obtained from the China Food Composition Database [9]. The validity and reproducibility of the FFQ has been reported previously; it is a useful tool to estimate B vitamins in the Chinese population [28–30]. The FFQ used in our study was slightly revised by specialists from the National Institute for Nutrition and Health, Chinese Center for Disease Control and Prevention, and was validated by a pilot study within our cohort.

#### Covariates

Sociodemographic information included age at study baseline, gender, education (in years), and residential status (categorized as solitude or not). Lifestyle factors included smoking status (determined by self-report and dichotomized as current smoker or not). Risk factors for cognitive decline included body mass index (the ratio of weight to squared height, BMI), diabetes (fasting glucose ≥ 7.0 mmol/L or antidiabetic medication), hypertension (measured blood pressure > 140/90 mmHg or antihypertensive medication), and coronary heart disease (CHD).

### Laboratory analysis

The blood samples for biochemical parameters were drawn via vein puncture after an overnight fast into a tube containing coagulant. The tube was then immediately centrifuged at 3000 g for 8 min at 4 °C, and the serum samples were collected and stored at − 80 °C until further analysis. Serum levels of B vitamins and metabolites (folate, vitamin B_6_, vitamin B_12_, and Hcy) were measured using an Abbott Architect i2000 immunoassay analyzer. Concentrations of oxidative biomarkers, including reactive oxygen species (ROS), malondialdehyde (MDA), 8-hydroxy-deoxyguanosine (8-OHdG), and 8-iso-prostaglandin F2α (8-iso-PGF2α), were measured using commercially available ELISA kits from Nanjing Jiancheng Biotechnology Institute Co., Ltd. according to the manufacturer's instructions.

### Genome-wide DNA methylation discovery and validation

Genomic DNA was isolated from peripheral blood leukocytes using the QIAamp DNA Blood Kit (Qiagen, CA, USA) and subjected to bisulfite conversion using the EZ DNA Methylation Kit (Zymo Research, CA, USA). Genome-wide DNA methylation using Illumina Infinium Human Methylation450K BeadChip was performed following the methylation protocol. DNA methylation was validated using MassArray® EpiTyper™ in BioMiao Biological Technology, Beijing, China. This method applied matrix-assisted laser desorption ionization time-of-flight (MALDI-TOF) mass spectrometry, of which the mass spectra were collected using a MassArray Compact MALDI-TOF, and the spectra’s methylation ratios were performed with EpiTYPER software (Sequenom, San Diego, CA). The design of polymerase chain reaction (PCR) primers covering 25 CpG sites of the NUDT15 gene and 17 CpG sites of the TXNRD1 gene and calculation of DNA methylation ratio were both done using Epidesigner software. The β value, representing methylation status of the individual probes, was used to indicate the methylation level at each CpG site, with the range from 0 (fully unmethylated) to 1 (fully methylated).

### Statistical analysis

Statistical analyses were performed using STATA version 13.0 (STATA, College Station, TX). Prior to analysis, the normality of the data distribution was checked. Continuous variables are expressed as median (interquartile ranges, IQR) when nonnormally distributed or as mean ± standard deviation (SD) when normally distributed. Student’s *t* test or the Mann–Whitney *U* test was used to compare continuous variables, as appropriate. The differences in the frequencies of the categorical variables were evaluated using the chi-square test or Fisher’s exact test. Longitudinal associations of dietary intakes of B vitamins with global and domain-specific cognitive decline were estimated using linear mixed-effect models. Dietary intake of vitamin B_6_, folate, and vitamin B_12_ were categorized into quartiles, and the quartile nearest the Chinese recommended nutrient intake (RNI, 2013 edition) was considered the reference. Spearman’s or Pearson’s correlation coefficient was calculated to analyze the relationships between DNA methylation levels and the biochemical values of participants. Moreover, multiple linear regression models were used to examine the effects of differentially methylated CpG sites and their interaction with B vitamins on cognitive performance in the validation group. All of the models were adjusted for covariates. A two-sided *P* < 0.05 was considered statistically significant, and issues of multiple testing were taken into account by considering *P* values adjusted for the false discovery rate (FDR).

## Results

### Characteristics of the sample

The study participants were 59 years old on average (range 50–70) at the time of the dietary survey, and 58.1% were female (Table 1). Baseline characteristics, including lifestyle, medical history of hypertension, diabetes, CHD, and smoking and drinking status, are also provided in Table 1. The median daily intakes were 2.3 mg/day for vitamin B_6_, 388.1 μg/day for folate, and 2.1 μg/day for vitamin B_12_. Compared with RNIs for the Chinese population aged 50–70 (vitamin B_6_: 1.6 mg/day; folate: 400 μg/day; vitamin B_12_: 2.4 μg/day), the average intakes of folate and vitamin B_12_ were inadequate in the study participants.

**Table 1:** Baseline characteristics and B vitamin intakes of the participants from EMCOA study (*n* = 2533)

Baseline characteristics	Overall sample	Men	Women
	(*N* = 2533)	(*N* = 1165)	(*N* = 1368)
Demographic characteristics			
Age	59 (55, 62)	59 (56, 63)	58 (55, 62)
Education years	9 (9, 12)	12 (9, 12)	9 (9, 12)
BMI (kg/m^2^)	24.5 (22.7, 26.6)	24.9 (23.2, 27.0)	24.2 (22.3, 26.2)
Lifestyle			
Current smoker, *n*(%)	597 (25.4%)	562 (48.2%)	35 (2.6%)
Current drinker, *n*(%)	634 (26.9%)	560 (48.1%)	74 (5.4%)
Medical history			
Diabetes, *n*(%)	381 (15.0%)	215 (18.5%)	166 (12.1%)
Hypertension, *n*(%)	834 (32.9%)	425 (36.5%)	409 (29.9%)
Coronary heart disease, *n*(%)	250 (9.9%)	148 (12.7%)	102 (7.5%)
Dietary B vitamin intakes			
Vitamin B_6_ (mg/day)	2.3 (1.8, 3.0)	2.4 (1.9, 3.3)	2.2 (1.7~2.8)
1st quartile	0.4~1.8	0.5~1.9	0.4~1.7
2nd quartile	1.8~2.3	1.9~2.4	1.7~2.2
3rd quartile	2.3~3.0	2.4~3.3	2.2~2.8
4th quartile	3.0~17.4	3.3~17.4	2.8~9.5
Folate (μg/day)	388.1 (274.8~534.2)	394.8 (277.5, 551.5)	381.1 (271.9, 518.9)
1st quartile	27.0~274.8	31.5~277.5	27.0~271.1
2nd quartile	274.8~388.1	277.5~394.8	271.1~381.1
3rd quartile	388.1~534.2	394.8~551.5	381.1~518.9
4th quartile	534.2~5952.2	551.5~5952.2)	518.9~3132.2
Vitamin B_12_ (μg/day)	2.1 (1.3, 3.4)	2.5 (1.6, 4.0)	1.9 (1.1, 2.8)
1st quartile	0~1.3	0~1.6	0~1.1
2nd quartile	1.3~2.1	1.6~2.5	1.1~1.9
3rd quartile	2.1~3.4	2.5~4.0	1.9~2.8
4th quartile	3.4~36.5	4.0~36.5	2.8~23.8

*BMI* body mass index

### Effects of B vitamin intake on cognitive decline

The results of fully adjusted mixed-effect linear regression analyses for B vitamin intakes and cognitive decline are shown in Tables 2, 3, and 4. Dietary intake of B vitamins was divided into four quartiles as categorical variables. Multivariate analyses adjusting for confounders took the quartile of each B vitamin nearest the RNI as the reference. Therefore, the 1st quartile of vitamin B_6_, 3rd quartile of folate, and 3rd quartile of vitamin B_12_ were used as references. As shown in Table 2, adequate intake of vitamin B_6_ higher than the RNI was significantly associated with better performance of verbal memory. However, the 4th quartile of vitamin B_6_ was negatively associated with the MoCA and DSF scores, suggesting that vitamin B_6_ intake much higher than the RNI may have adverse effects on global cognition and attention. Additionally, adequate intake of folate higher than the RNI was significantly associated with better cognitive reserve for global cognition, verbal memory, and attention, whereas no associations were observed between inadequate intake of folate and cognitive decline (Table 3). In contrast, severe deficiency of vitamin B_12_ (1st quartile) was significantly associated with accelerated cognitive decline across all domains, and moderate deficiency (2nd quartile) was associated with decline in most cognitive domains. Beneficial effects of adequate vitamin B_12_ intake on global cognitive reserve were observed in both the MMSE and MoCA scores (Table 4).

**Table 2:** Longitudinal associations of dietary intake of vitamin B_6_ with global and domain-specific cognitive decline

Cognitive performance		Vitamin B_6_ (mg/day)			
		1st quartile	2nd quartile	3rd quartile	4th quartile
MMSE	*B* (95%CI)	Ref	− 0.04 (− 0.17, 0.10)	− 0.13 (− 0.29, 0.02)	− 0.07 (− 0.27, 0.13)
	*P* value	Ref	0.586	0.091	0.499
MoCA	*B* (95%CI)	Ref	− 0.06 (− 0.27, 0.14)	− 0.26 (− 0.56, 0.04)	− 0.31 (− 0.55, − 0.08)
	*P* value	Ref	0.555	0.094	0.009*
AVLT-IR	*B* (95%CI)	Ref	0.35 (0.03, 0.68)	0.40 (0.03, 0.77)	0.66 (0.18, 1.14)
	*P* value	Ref	0.033*	0.034*	0.007*
AVLT-SR	*B* (95%CI)	Ref	0.33 (0.17, 0.50)	0.30 (0.12, 0.49)	0.46 (0.22, 0.70)
	*P* value	Ref	< 0.001*	0.002*	< 0.001*
AVLT-LR	*B* (95%CI)	Ref	0.25 (0.07, 0.43)	0.39 (0.12, 0.65)	0.19 (− 0.02, 0.40)
	*P* value	Ref	0.007*	0.004*	0.069
SDMT	*B* (95%CI)	Ref	1.13 (0.44, 1.82)	0.76 (− 0.03, 1.55)	− 0.22 (− 1.23, 0.79)
	*P* value	Ref	0.001*	0.058	0.669
LMT	*B* (95%CI)	Ref	0.20 (− 0.24, 0.63)	− 0.06 (− 0.56, 0.43)	− 0.16 (− 0.80, 0.48)
	*P* value	Ref	0.376	0.799	0.625
DSF	*B* (95%CI)	Ref	− 0.02 (− 0.11, 0.07)	− 0.04 (− 0.15, 0.06)	− 0.17 (− 0.30, − 0.04)
	*P* value	Ref	0.664	0.427	0.012*
DSB	*B* (95%CI)	Ref	0.03 (− 0.06, 0.11)	− 0.03 (− 0.12, 0.07)	− 0.05 (− 0.17, 0.07)
	*P* value	Ref	0.512	0.604	0.426

*MMSE* mini-mental state examination, *MoCA* Montreal Cognitive Assessment, *AVLT*-*IR* auditory verbal learning test-immediate recall, *AVLT*-*SR* auditory verbal learning test-short recall, *AVLT*-*LR* auditory verbal learning test-long recall, *SDMT* symbol digit modalities test, *LMT* logical memory test, *DSF* digit span forwards, *DSB* digit span backwards

*B*: regression coefficients of linear mixed-effect models

^∗^*P* < 0.05

**Table 3:** Longitudinal associations of dietary intake of folate with global and domain-specific cognitive decline

Cognitive performance		Folate (μg/day)			
		1st quartile	2nd quartile	3rd quartile	4th quartile
MMSE	*B* (95%CI)	− 0.11 (− 0.25, 0.04)	0.09 (− 0.05, 0.23)	Ref	0.06 (− 0.09, 0.22)
	*P* value	0.155	0.188	Ref	0.418
MoCA	*B* (95%CI)	− 0.17 (− 0.40, 0.06)	− 0.04 (− 0.25, 0.17)	Ref	0.25 (0.01, 0.48)
	*P* value	0.14	0.732	Ref	0.039*
AVLT-IR	*B* (95%CI)	0.32 (− 0.04, 0.67)	0.09 (− 0.24, 0.42)	Ref	− 0.07 (− 0.44, 0.30)
	*P* value	0.081	0.604	Ref	0.7
AVLT-SR	*B* (95%CI)	0.07 (− 0.1, 0.24)	0.01 (− 0.18, 0.20)	Ref	0.23 (0.05, 0.41)
	*P* value	0.405	0.939	Ref	0.013*
AVLT-LR	*B* (95%CI)	0.12 (− 0.07, 0.32)	− 0.10 (− 0.29, 0.08)	Ref	− 0.01 (− 0.21, 0.20)
	*P* value	0.219	0.286	Ref	0.966
SDMT	*B* (95%CI)	0.57 (− 0.18, 1.33)	0.56 (− 0.14, 1.27)	Ref	0.51 (− 0.28, 1.30)
	*P* value	0.138	0.119	Ref	0.207
LMT	*B* (95%CI)	− 0.03 (− 0.51, 0.45)	0.01 (− 0.44, 0.46)	Ref	0.60 (0.10, 1.10)
	*P* value	0.903	0.963	Ref	0.019*
DSF	*B* (95%CI)	− 0.01 (− 0.11, 0.08)	− 0.07 (− 0.16, 0.02)	Ref	0.12 (0.02, 0.22)
	*P* value	0.771	0.138	Ref	0.023*
DSB	*B* (95%CI)	− 0.01 (− 0.09, 0.09)	− 0.03 (− 0.12, 0.06)	Ref	0.05 (− 0.04, 0.15)
	*P* value	0.997	0.497	Ref	0.266

*MMSE* mini-mental state examination, *MoCA* Montreal Cognitive Assessment, *AVLT*-*IR* auditory verbal learning test-immediate recall, *AVLT*-*SR* auditory verbal learning test-short recall, *AVLT*-*LR* auditory verbal learning test-long recall, *SDMT* symbol digit modalities test, *LMT* logical memory test, *DSF* digit span forwards, *DSB* digit span backwards

*B*: regression coefficients of linear mixed-effect models

^∗^*P* < 0.05

**Table 4:** Longitudinal associations of dietary intake of vitamin B_12_ with global and domain-specific cognitive decline

Cognitive performance		Vitamin B_12_ (μg/day)			
		1st quartile	2nd quartile	3rd quartile	4th quartile
MMSE	*B* (95%CI)	− 0.30 (− 0.43, − 0.16)	− 0.21 (− 0.34, − 0.08)	Ref	0.32 (0.17, 0.46)
	*P* value	< 0.001*	0.002*	Ref	< 0.001*
MoCA	*B* (95%CI)	− 0.56 (− 0.77, − 0.36)	− 0.49 (− 0.69, − 0.29)	Ref	0.38 (0.16, 0.60)
	*P* value	< 0.001*	< 0.001*	Ref	0.001*
AVLT-IR	*B* (95%CI)	− 0.65 (− 0.97, − 0.33)	− 0.60 (− 0.92, − 0.27)	Ref	− 0.01 (− 0.35, 0.35)
	*P* value	< 0.001*	< 0.001*	Ref	0.987
AVLT-SR	*B* (95%CI)	− 0.30 (− 0.46, − 0.14)	− 0.08 (− 0.24, 0.09)	Ref	− 0.04 (− 0.21, 0.14)
	*P* value	< 0.001*	0.352	Ref	0.687
AVLT-LR	*B* (95%CI)	− 0.32 (− 0.49, − 0.14)	− 0.11 (− 0.29, 0.07)	Ref	0.01 (− 0.19, 0.20)
	*P* value	< 0.001*	0.231	Ref	0.957
SDMT	*B* (95%CI)	− 1.78 (− 2.47, − 1.09)	− 1.22 (− 1.89, − 0.55)	Ref	1.17 (− 0.43, 1.92)
	*P* value	< 0.001*	< 0.001*	Ref	0.272
LMT	*B* (95%CI)	− 0.70 (− 1.14, − 0.26)	− 0.69 (− 1.12, − 0.27)	Ref	0.17 (− 0.30, 0.64)
	*P* value	0.002*	0.001*	Ref	0.483
DSF	*B* (95%CI)	− 0.13 (− 0.22, − 0.03)	0.01 (− 0.08, 0.09)	Ref	− 0.08 (− 0.18, 0.02)
	*P* value	0.007*	0.893	Ref	0.117
DSB	*B* (95%CI)	− 0.11 (− 0.20, − 0.03)	− 0.11 (− 0.19, − 0.03)	Ref	0.03 (− 0.06, 0.12)
	*P* value	0.007*	0.008*	Ref	0.487

*MMSE* mini-mental state examination, *MoCA* Montreal Cognitive Assessment, *AVLT*-*IR* auditory verbal learning test-immediate recall, *AVLT*-*SR* auditory verbal learning test-short recall, *AVLT*-*LR* auditory verbal learning test-long recall, *SDMT* symbol digit modalities test, *LMT* logical memory test, *DSF* digit span forwards, *DSB* digit span backwards

*B*: regression coefficients of linear mixed-effect models

^∗^*P* < 0.05

### Comparison of selected MCI patients and controls in the discovery and validation groups

**Fig. 2:**
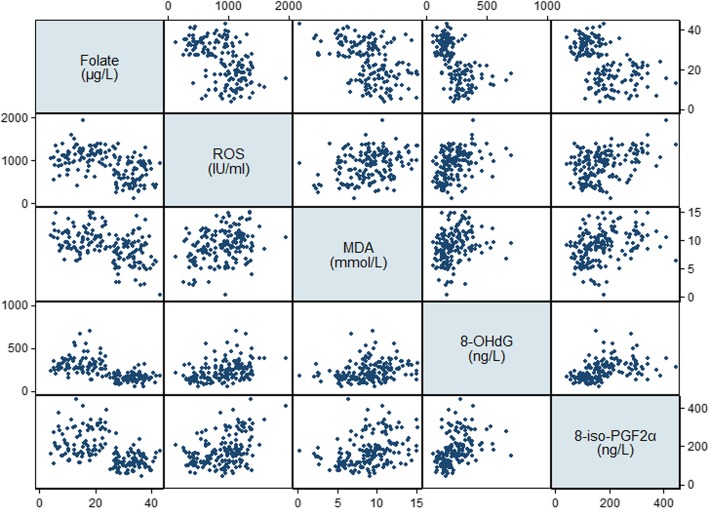
Scatterplot matrix of serum folate and oxidative biomarkers. *ROS* reactive oxygen species, *MDA* malondialdehyde, 8-*OHdG* 8-hydroxy-desoxyguanosine, 8-*iso*-*PGF2α* 8-iso-prostaglandin F2α

**Fig. 3:**
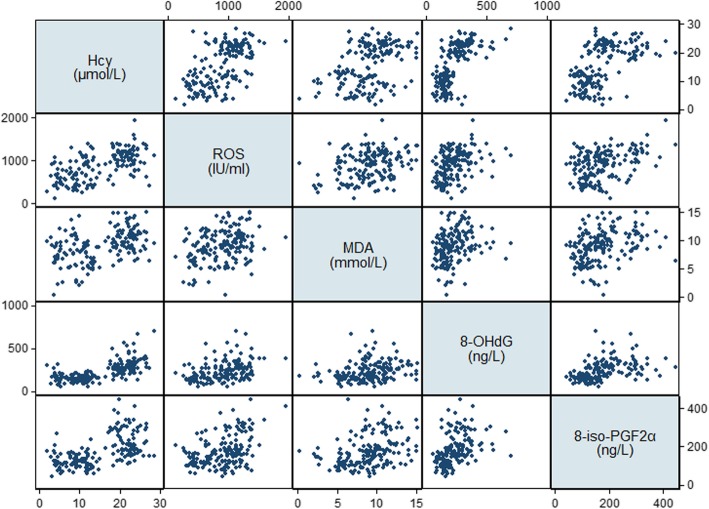
Scatterplot matrix of serum Hcy and oxidative biomarkers. *Hcy* homocysteine, *ROS* reactive oxygen species, *MDA* malondialdehyde, 8-*OHdG* 8-hydroxy-desoxyguanosine, 8-*iso*-*PGF2α* 8-iso-prostaglandin F2α

The general characteristics, cognitive performance, dietary intakes, and serum levels of B vitamins and oxidative biomarkers for selected MCI patients and cognitively normal controls were determined (Table 5). In the discovery group, there were no significant differences in sociodemographic or lifestyle factors between MCI patients and controls, whereas MCI patients in the validation group were more likely to be male (*P* = 0.041) and less likely to be current drinkers (*P* < 0.001). With respect to cognitive performance, the scores of MoCA (*P* = 0.005), LMT (*P* = 0.010), and DSF (*P* = 0.010) and serum levels of folate (*P*=0.003) were significantly lower, whereas levels of Hcy (*P* < 0.001) and 8-iso-PGF2α (*P* = 0.049) were significantly higher, in MCI patients in the discovery group. These significant differences were replicated in the validation group in addition to the finding that all of the domain-specific cognitive scores and dietary intake of folate were significantly lower, whereas all of the oxidative biomarkers were significantly higher, in MCI patients (*P* < 0.05). Significant negative correlations were observed between folate and oxidative biomarkers, whereas Hcy was positively correlated with oxidative biomarkers (*P* < 0.001, Table 6, Figs. 2 and 3). Overall, MCI patients tended to have folate deficiency, disturbances of B vitamin metabolism, and imbalanced redox status.

**Table 5:** General characteristics, cognitive performance, serum B vitamin biomarkers and oxidative biomarkers of study populations for DNA methylation discovery and validation

	Human Methylation450K BeadChip for discovery group			MassARRAY EpiTYPER for validation group		
	MCI	Controls	FDR adjusted *P* value	MCI	Controls	FDR adjusted *P* value
	*N* = 7	*N* = 5		*N* = 102	*N* = 68	
General characteristics						
Age	60.0 ± 2.4	61.4 ± 2.9	0.481	59.6 ± 3.0	59.6 ± 3.4	1.000
Gender (M/F)	3/4	2/3	1	40/62	40/28	0.041*
Education years	9.1 ± 3.3	10.8 ± 1.6	0.474	9 (9, 12)	11 (9, 15)	0.582
BMI (kg/m^2^)	26.0 ± 2.3	24.1 ± 2.4	0.386	24.2 ± 2.5	25.0 ± 2.7	0.134
Current smoker, *n*(%)	3 (42.9%)	2 (40.0%)	1	25 (24.5%)	25 (36.8%)	0.226
Current drinker, *n*(%)	1 (14.3%)	2 (40.0%)	0.572	19 (18.6%)	32 (47.1%)	< 0.001*
Diabetes, *n*(%)	3 (42.9%)	0 (0.0%)	0.407	20 (19.6%)	8 (11.8%)	0.375
Hypertension, *n*(%)	3 (42.9%)	2 (40.0%)	1	31 (31.4%)	22 (32.3%)	0.842
CHD, *n*(%)	2 (28.6%)	0 (0.0%)	0.416	5 (4.9%)	2 (2.9%)	0.691
Cognitive performance						
MMSE	28.0 (24.0, 29.0)	28.0 (29.0, 29.0)	0.411	28.0 (27.0, 29.0)	28.5 (27.0, 30.0)	0.226
MoCA	20.3 ± 2.8	26.6 ± 0.9	0.005*	22.4 ± 3.0	26.1 ± 2.3	< 0.001*
AVLT-IR	13.3 ± 4.1	16.8 ± 2.8	0.323	15.3 ± 4.5	18.4 ± 4.7	< 0.001*
AVLT-SR	4.6 ± 3.6	6.4 ± 1.5	0.474	5.1 ± 2.7	6.4 ± 2.3	0.003*
AVLT-LR	3.9 ± 4.0	3.8 ± 2.7	0.96	4 (2, 7)	6 (3, 8)	0.007*
SDMT	30.4 ± 14.3	41.2 ± 8.6	0.336	33 (25, 40)	37 (31, 46)	0.015*
LMT	8.0 ± 3.6	16.2 ± 3.1	0.010*	10.0 (5.5, 13.0)	14.5 (10.0, 16.5)	< 0.001*
DSF	6.5 ± 1.0	8.8 ± 0.8	0.010*	7.2 ± 1.3	7.9 ± 1.5	0.008*
DSB	4.3 ± 1.0	4.8 ± 0.4	0.474	4 (3, 5)	4 (4, 5)	0.016*
Dietary B vitamin intakes						
Vitamin B_6_ (mg/day)	1.9 (1.4, 2.0)	2.6 (1.5, 2.8)	0.596	2.2 (1.8, 2.6)	2.2 (1.7, 2.7)	0.720
Folate (μg/day)	311.6 (203.6, 496.4)	577.2 (239.4, 705.0)	0.596	307.5 (242.6, 442.7)	376.7 (271.8, 532.5)	0.019*
Vitamin B_12_ (μg/day)	1.2 (0.3, 2.8)	3.2 (1.8, 3.9)	0.427	2.0 (1.3, 2.9)	2.0 (1.2, 3.1)	0.936
Serum B vitamin biomarkers						
Vitamin B_6_ (ng/L)	650.3 ± 125.0	542.3 ± 108.5	0.326	618.6 (531.2, 709.2)	596.5 (514.0, 645.9)	0.193
Folate (μg/L)	15.0 ± 5.6	30.2 ± 3.7	0.003*	17.4 (11.2, 22.8)	32.2 (28.0, 35.6)	< 0.001*
Vitamin B_12_ (ng/L)	184.0 (113.0, 305.0)	284.0 (216.0, 333.5)	0.573	211.5 (174.8, 262.9)	198.5 (143.5, 256.3)	0.321
Hcy (μmol/L)	22.5 ± 2.9	9.8 ± 2.8	< 0.001*	21.1 (18.3, 23.4)	9.4 (6.7, 11.9)	< 0.001*
Oxidative biomarkers						
ROS (IU/ml)	1000.8 ± 345.3	779.1 ± 424.8	0.474	1043.8 (780.2, 1209.5)	728.0 (493.8, 964.8)	< 0.001*
MDA (mmol/L)	10.0 ± 1.7	7.0 ± 3.5	0.225	9.7 ± 2.8	8.1 ± 2.6	0.003*
8-OHdG (ng/L)	338.1 ± 112.2	185.2 ± 46.3	0.061	271.0 (201.1, 341.1)	151.2 (125.9, 176.7)	< 0.001*
8-iso-PGF2α (ng/L)	221.6 ± 59.1	118.2 ± 56.22	0.049*	188.0 (152.0, 279.6)	114.0 (90.3, 144.3)	< 0.001*

*MMSE* mini-mental state examination, *MoCA* Montreal Cognitive Assessment, *AVLT*-*IR* auditory verbal learning test-immediate recall, *AVLT*-*SR* auditory verbal learning test-short recall, *AVLT*-*LR* auditory verbal learning test-long recall, *SDMT* symbol digit modalities test, *LMT* logical memory test, *DSF* digit span forwards, *DSB* digit span backwards, *BMI* body mass index, *CHD* coronary heart disease, *Hcy* homocysteine, *ROS* reactive oxygen species, *MDA* malondialdehyde, 8-*OHdG* 8-hydroxy-desoxyguanosine, 8-*iso*-*PGF2α* 8-iso-prostaglandin F2α, *FDR* false discovery rate

Data shown as median (interquartile range) were compared between two groups using the Mann—Whitney *U* test

Data shown as mean ± standard deviation were compared between two groups using the Student *t* test

Data shown as *n* (%) were compared between two groups using the chi-square test or Fisher’s exact test

^∗^*P* < 0.05

**Table 6:** Correlations between serum B vitamin biomarkers and oxidative biomarkers

	Oxidative biomarkers							
	ROS (IU/ml)		MDA (mmol/L)		8-OHdG (ng/L)		8-iso-PGF2α (ng/L)	
	*r*	FDR adjusted *P* value	*r*	FDR adjusted *P* value	*r*	FDR adjusted *P* value	*r*	FDR adjusted *P* value
Serum B vitamin biomarkers								
Folate (μg/L)	− 0.507	< 0.001*	− 0.432	< 0.001*	− 0.653	< 0.001*	− 0.568	< 0.001*
Vitamin B_6_ (ng/L)	0.069	0.742	0.155	0.293	− 0.008	0.991	0.194	0.223
Vitamin B_12_ (ng/L)	0.114	0.588	0.109	0.588	0.220	0.097	0.177	0.194
Hcy (μmol/L)	0.564	< 0.001*	0.400	< 0.001*	0.666	< 0.001*	0.547	< 0.001*

*Hcy* homocysteine, *ROS* reactive oxygen species, *MDA* malondialdehyde, 8-*OHdG* 8-hydroxy-desoxyguanosine, 8-*iso*-*PGF2α* 8-iso-prostaglandin F2α, *FDR* false discovery rate

^∗^*P* < 0.05

**Fig. 4:**
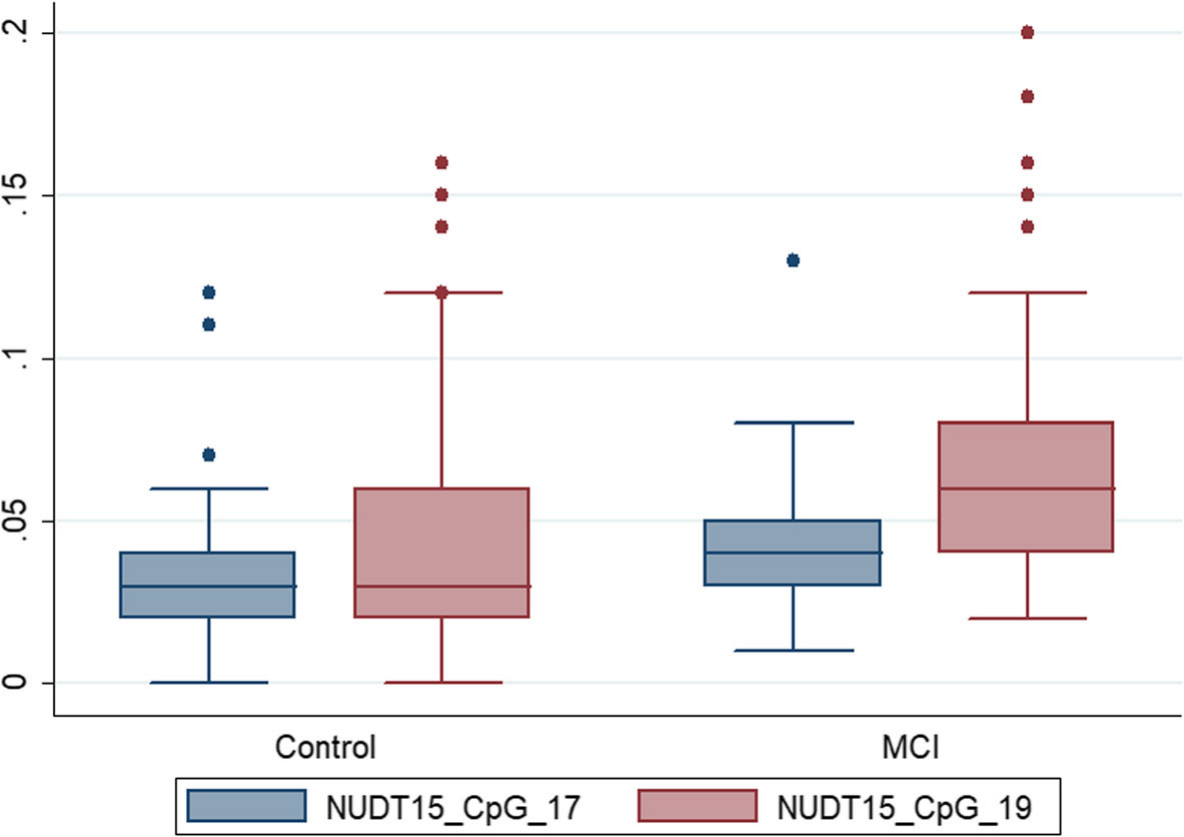
Comparisons in DNA methylation for CpG_17 and CpG_19 located within NUDT15 between MCI patients and controls. The box lines represent median ± interquartile ranges of DNA methylation, which is expressed as a beta value (0-1). *NUDT15* nudix hydrolase 15, *TXNRD1* thioredoxin reductase 1, *MCI* mild cognitive impairment

**Fig. 5:**
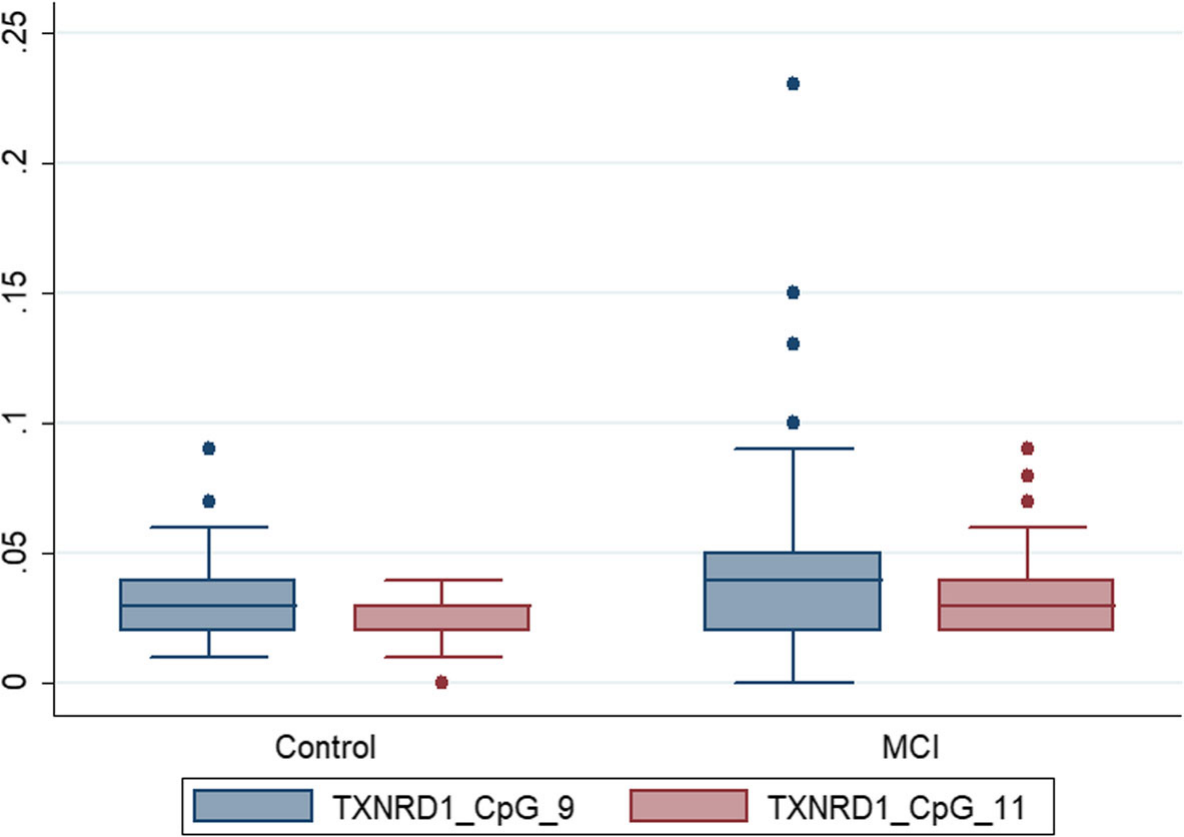
Comparisons in DNA methylation for CpG_9 and CpG_11 located within TXNRD1 between MCI patients and controls. The box lines represent median ± interquartile ranges of DNA methylation, which is expressed as a beta value (0-1). *NUDT15* nudix hydrolase 15, *TXNRD1* thioredoxin reductase 1, *MCI* mild cognitive impairment

A total of 2277 differentially methylated CpG sites were identified in the discovery group when comparing MCI cases (*n* = 7) versus controls (*n* = 5). The associations between DNA methylation levels of the oxidative stress-related genes and serum B vitamin biomarkers were analyzed. Negative correlations were found between the serum level of folate and DNA methylation levels of CpG sites in the nudix hydrolase 15 (NUDT15) and thioredoxin reductase 1 (TXNRD1) genes. The significant CpG sites were located in TSS1500 for NUDT15 and the 5′UTR for TXNRD1. Forty-two CpG candidate sites located in these genes were selected for further validation. The methylation of CpG_17 and CpG_19 in NUDT15 as well as CpG_9 CpG_11 in TXNRD1 showed significant differences between MCI patients and controls (FDR adjusted *P* < 0.05, Table 7, Figs. 4 and 5).

**Table 7:** Variation of the methylation patterns of CpG sites located in NUDT15 and TNXRD1 from validation

Gene	Gene feature	CpG	Sites	Fold change in methylation (MCI vs. control)	FDR adjusted *P* value
NUDT15	TSS1500	cg01596986	CpG_1	0.751	0.586
			CpG_2	0.955	0.714
			CpG_3	0.955	0.714
			CpG_4	0.795	0.089
			CpG_5	1.180	0.122
			CpG_6	0.649	0.842
			CpG_7	1.078	0.973
			CpG_8	0.992	0.975
			CpG_9	1.105	0.910
			CpG_10	2.351	0.582
			CpG_11	1.048	0.476
			CpG_12	1.062	0.661
			CpG_13	0.919	0.582
			CpG_14	1.026	0.810
			CpG_15	0.959	0.935
			CpG_16	1.088	0.447
			CpG_17	1.157	0.030*
			CpG_18	1.210	0.525
			CpG_19	1.288	0.024*
			CpG_20	0.952	0.529
			CpG_21	1.210	0.525
			CpG_22	1.245	0.276
			CpG_23	1.022	0.935
			CpG_24	1.092	0.594
			CpG_25	1.119	0.529
TXNRD1	5′UTR	cg12166806	CpG_1	0.917	0.714
			CpG_2	0.787	0.615
			CpG_3	1.495	0.375
			CpG_4	1.092	0.375
			CpG_5	0.941	0.890
			CpG_6	1.092	0.375
			CpG_7	1.156	0.447
			CpG_8	1.217	0.386
			CpG_9	1.015	0.029*
			CpG_10	0.987	0.910
			CpG_11	1.191	0.018*
			CpG_12	1.120	0.910
			CpG_13	0.982	0.975
			CpG_14	0.940	0.475
			CpG_15	1.139	0.241
			CpG_16	1.054	0.626
			CpG_17	1.310	0.475

*FDR* false discovery rate, *NUDT15* nudix hydrolase 15, *TXNRD1* thioredoxin reductase 1, *TSS1500* 1500 base pairs around the transcription start site, 5′*UTR* 5’untranslated region

∗*P*<0.05

### Analysis of correlations and regression models

**Fig. 6:**
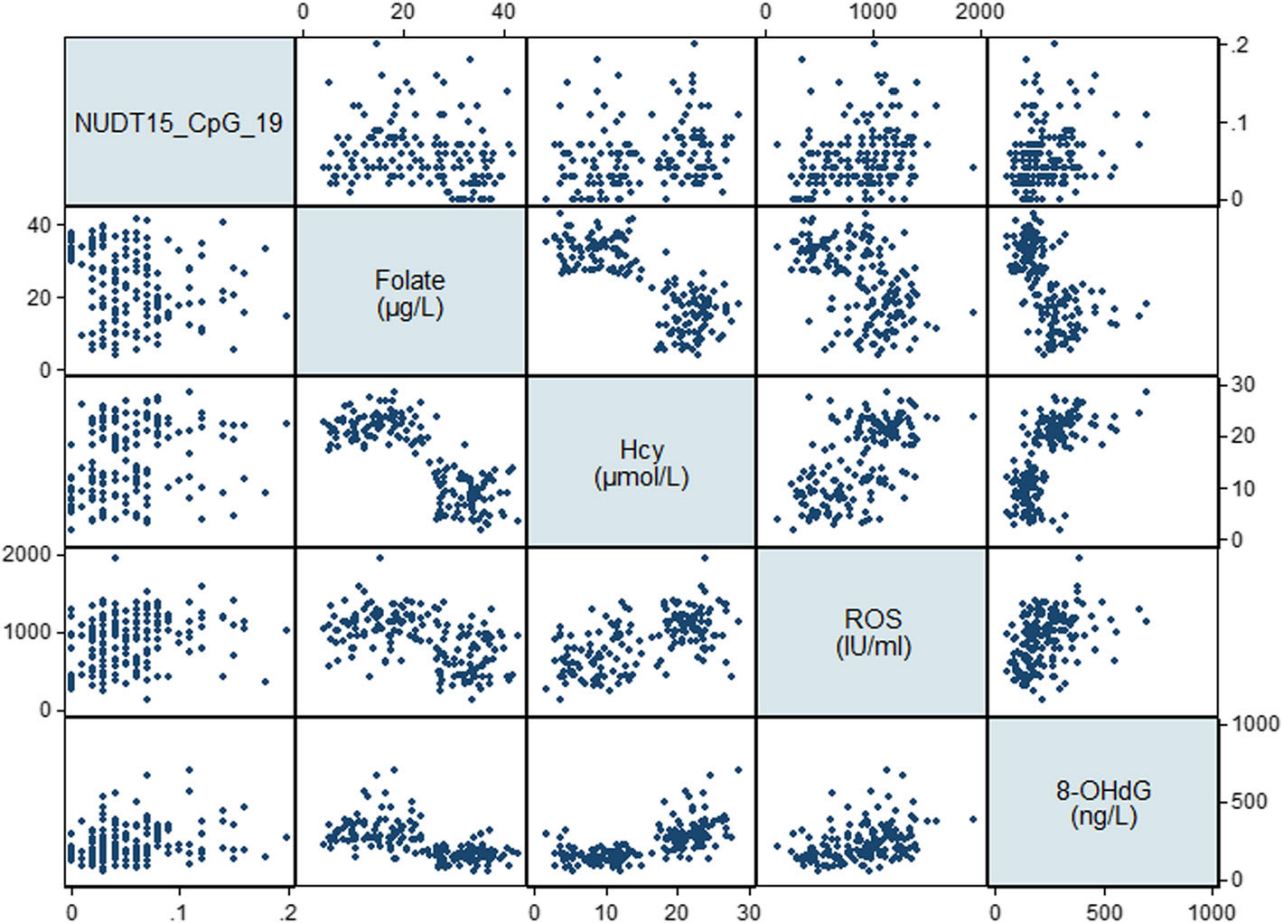
Scatterplot matrix of B vitamin and oxidative biomarkers correlated with DNA methylation levels of NUDT15. *NUDT15* nudix hydrolase 15, *Hcy* homocysteine, *ROS* reactive oxygen species, 8-*OHdG* 8-hydroxy-desoxyguanosine

**Fig. 7:**
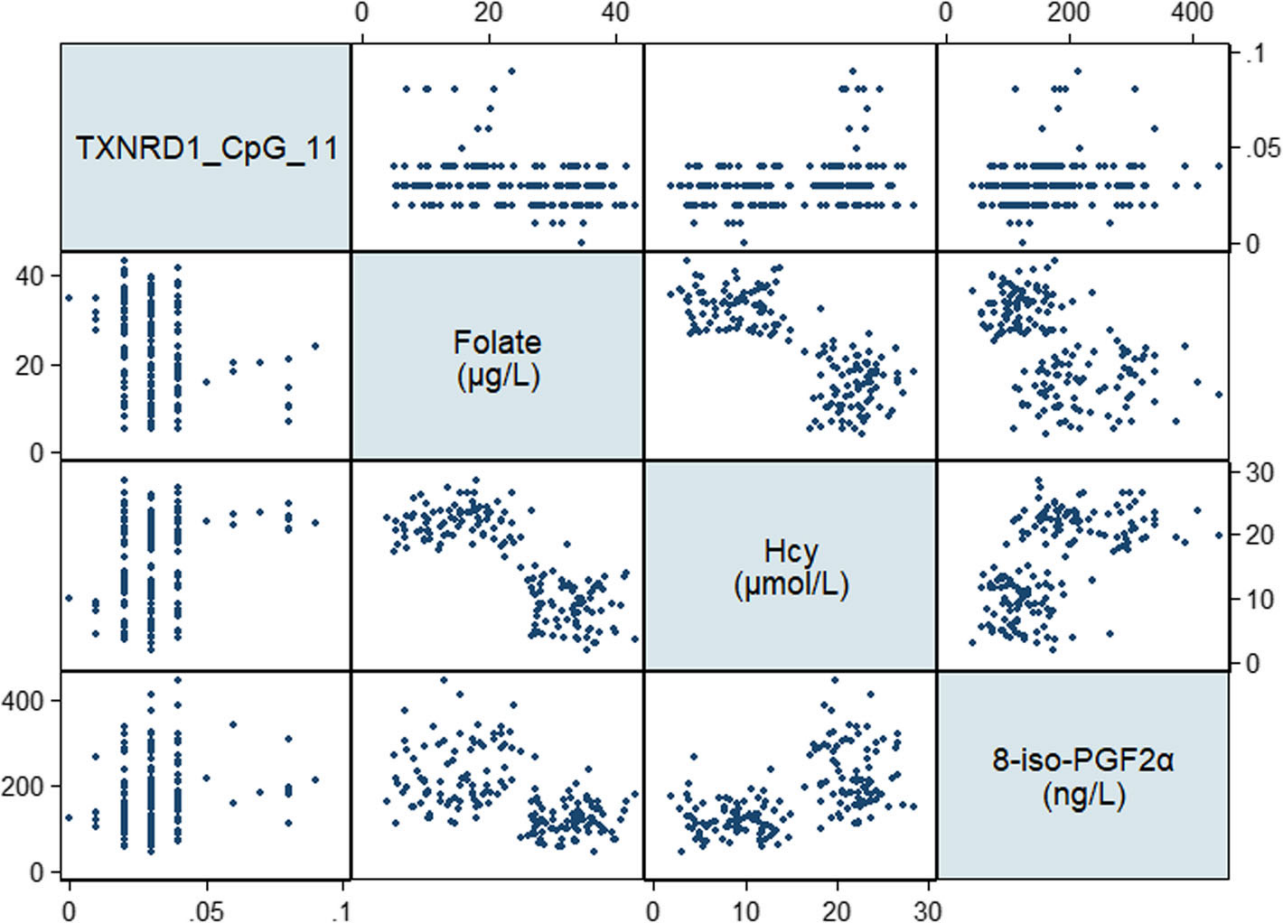
Scatterplot matrix of B vitamin and oxidative biomarkers correlated with DNA methylation levels of TXNRD1. *TXNRD1* thioredoxin reductase 1, *Hcy* homocysteine, 8-*iso*-*PGF2α* 8-iso-prostaglandin F2α

Table 8 demonstrates that the DNA methylation levels of CpG_ 19 of NUDT15 and CpG_11 of TXNRD1 had significantly negative correlations with folate (*r* = − 0.219, FDR adjusted *P* = 0.028 for NUDT15; *r* = − 0.192, FDR adjusted *P* = 0.041 for TXNRD1) and positive correlations with Hcy (*r* = 0.251, FDR adjusted *P* = 0.010 for NUDT15; *r* = 0.225, FDR adjusted *P* = 0.045 for TXNRD1). In addition, CpG_ 19 of NUDT15 had significant correlations with ROS (*r* = 0.230, FDR adjusted *P* = 0.020) and 8-OHdG (*r* = 0.259, FDR adjusted *P* = 0.008) and CpG_11 of TXNRD1 with 8-iso-PGF2α (*r* = 0.192, FDR adjusted *P* = 0.042, Table 8, Figs. 6 and 7).

**Table 8:** Correlations for serum B vitamin biomarkers, oxidative biomarkers, and DNA methylation of NUDT15 and TXNRD1 genes

	NUDT15_CpG_17		NUDT15_CpG_19		TXNRD1_CpG_9		TXNRD1_CpG_11	
	*r*	FDR adjusted *P* value	*r*	FDR adjusted *P* value	r	FDR adjusted *P* value	*r*	FDR adjusted *P* value
B vitamin and metabolites								
Folate (μg/L)	− 0.101	0.417	− 0.219	0.028*	− 0.201	0.133	− 0.192	0.041*
Vitamin B_6_ (ng/L)	− 0.030	0.815	− 0.074	0.559	0.009	0.949	0.029	0.930
Vitamin B_12_ (ng/L)	0.022	0.962	0.041	0.930	0.086	0.738	0.079	0.738
Hcy (μmol/L)	0.098	0.434	0.251	0.010*	0.198	0.133	0.225	0.045*
Oxidative biomarkers								
ROS (IU/ml)	0.069	0.762	0.230	0.020*	0.164	0.169	0.157	0.176
MDA (mmol/L)	0.068	0.561	0.125	0.268	0.143	0.236	0.123	0.296
8-OHdG (ng/L)	0.132	0.248	0.259	0.008*	0.180	0.145	0.171	0.121
8-iso-PGF2α (ng/L)	0.130	0.248	0.183	0.074	0.154	0.210	0.192	0.042*

*Hcy* homocysteine, *ROS* reactive oxygen species, *MDA* malondialdehyde, 8-*OHdG* 8-hydroxy-desoxyguanosine, 8-*iso*-*PGF2α* 8-iso-prostaglandin F2α, *FDR* false discovery rate, *NUDT15* nudix hydrolase 15, *TXNRD1* thioredoxin reductase 1

^∗^*P* < 0.05

We used multiple linear regression models to investigate the associations of B vitamin-related hypermethylated CpG sites in NUDT15 and TXNRD1 with cognitive performance (Table 9). These analyses allowed us to test whether hypermethylated CpG sites in NUDT15 and TXNRD1 were each associated with global or domain-specific cognitive performance. Next, we investigated whether serum levels of folate, vitamin B_12_, and Hcy and hypermethylated CpG sites in NUDT15 and TXNRD1 were synergistically associated with cognitive performance. Synergistic effects were tested in models that included a two-way interaction term; this allowed us to assess whether the impact of B vitamins on cognitive performance was mediated by DNA methylation of NUDT15 or TXNRD1. These models were repeated for all of the cognitive tests. Analyses revealed no significant associations of NUDT15 or TXNRD1 with cognitive performance (all *P* > 0.05) but significant synergism between DNA methylation of NUDT15 and Hcy, TXNRD1 and folate, as well as TXNRD1 and Hcy, such that the combination of NUDT15 and Hcy was associated with lower scores of MoCA (*B* = − 0.325; *P* < 0.001), LMT (*B* = − 0.187; *P* = 0.015), and DSF (*B* = − 0.221; *P* < 0.005). The interaction between TXNRD1 and Hcy was also selectively associated with MoCA (*B* = − 0.345; *P* < 0.001), LMT (*B* = − 0.269; *P* < 0.001), and DSF (*B* = − 0.252; *P* = 0.001) in addition to SDMT (*B* = − 0.137; *P* = 0.049). In contrast, the DNA methylation of TXNRD1 and serum level of folate were synergistically associated with better cognitive performance on MMSE (*B* = 0.147; *P* = 0.048), MoCA (*B* = 0.268; *P* < 0.001), and AVLT-IR (*B* = 0.163; *P* = 0.042).

**Table 9:** Effects of DNA methylation and the interaction with B vitamin biomarkers on global and domain-specific cognitive performance

Cognitive performance		NUDT15	NUDT15×Folate	NUDT15×Hcy	TXNRD1	TXNRD1×Folate	TXNRD1×Hcy
MMSE	*B*	− 0.018	0.044	− 0.082	− 0.005	0.147	− 0.103
	*P* value	0.809	0.559	0.287	0.947	0.048*	0.178
MoCA	*B*	− 0.127	0.096	− 0.325	− 0.124	0.268	− 0.345
	*P* value	0.077	0.183	< 0.001*	0.082	< 0.001*	< 0.001*
AVLT-IR	*B*	− 0.026	0.102	− 0.125	− 0.123	0.163	− 0.259
	*P* value	0.752	0.208	0.130	0.126	0.042*	0.001*
AVLT-SR	*B*	0.013	0.066	− 0.055	− 0.082	0.022	− 0.151
	*P* value	0.870	0.39	0.489	0.283	0.775	0.056
AVLT-LR	*B*	− 0.004	0.063	− 0.062	− 0.054	0.090	− 0.151
	*P* value	0.960	0.421	0.442	0.487	0.251	0.060
SDMT	*B*	− 0.086	− 0.002	− 0.132	− 0.090	0.051	− 0.137
	*P* value	0.213	0.980	0.059	0.185	0.456	0.049*
LMT	*B*	− 0.046	0.098	− 0.187	− 0.129	0.091	− 0.269
	*P* value	0.549	0.194	0.015*	0.186	0.231	< 0.001*
DSF	*B*	− 0.087	0.034	− 0.221	− 0.095	0.106	− 0.252
	*P* value	0.262	0.664	0.005*	0.223	0.174	0.001*
DSB	*B*	− 0.064	− 0.003	− 0.079	0.020	0.118	− 0.052
	*P* value	0.410	0.969	0.314	0.797	0.127	0.509

*MMSE* mini-mental state examination, *MoCA* Montreal Cognitive Assessment, *AVLT*-*IR* auditory verbal learning test-immediate recall, *AVLT*-*SR* auditory verbal learning test-short recall, *AVLT*-*LR* auditory verbal learning test-long recall, *SDMT* symbol digit modalities test, *LMT* logical memory test, *DSF* digit span forwards, *DSB* digit span backwards, *Hcy* homocysteine, *NUDT15* nudix hydrolase 15, *TXNRD1* thioredoxin reductase 1

*B*: standardized regression coefficients of multiple linear regression

^∗^*P* < 0.05

## Discussion

In our prospective study including 2533 middle-aged and elderly Chinese persons who initially had normal cognitive performance, we found that dietary vitamin B_12_ deficiency was associated with a greater rate of cognitive decline, whereas adequate intakes of folate and vitamin B_6_ were correlated with better cognitive reserve, indicating that dietary intakes of B vitamins are important predictors of cognitive changes. In a secondary case-control analysis, MCI patients had a significantly higher level of Hcy, which may result from significantly lower intake and serum levels of folate. Such biomarker patterns were significantly associated with higher levels of oxidative biomarkers and DNA methylation levels of redox-related genes. The interactions of folate and Hcy with DNA methylation could influence cognitive performance. To our knowledge, this is the first study that combined a longitudinal and case-control design to consider the impact on cognitive health of both dietary intakes and biomarker statuses of B vitamins that are involved in DNA methylation and oxidative stress.

Folate, along with vitamins B_12_ and B_6_, is essential in one-carbon metabolism, a network of reactions involving the transfer of one-carbon units. In one-carbon metabolism, tetrahydrofolate obtains a carbon unit in a vitamin B_6_-dependent reaction forming 5,10-methylenetetrahydrofolate, which is then converted to 5-methyltetrahydrofolate. 5-Methyltetrahydrofolate donates its methyl group to homocysteine in a reaction that uses vitamin B_12_ as a cofactor. Deficiencies in any of these B vitamins can perturb this complex regulatory network, resulting in hyperhomocysteinemia, which has been demonstrated to be a causal contributor to cognitive decline, MCI, and AD by extensive epidemiological studies in healthy older adults and patient populations [31]. Further research has investigated the relative importance of each of the B vitamins and Hcy for developing MCI and AD. Quadri et al. [32] have reported that participants with the highest Hcy levels (> 14.6 μmol/L) and the lowest folate levels (< 13.5 nmol/L) were more than three times likelier to develop AD. In addition, Ramos et al. [33] demonstrated that increased folate concentrations could significantly decrease the risk of developing dementia. To further complicate matters, Hann et al. [34] reported that Hcy is associated with a greater risk of dementia or cognitive impairment without dementia and that higher B_12_ concentrations may reduce this risk. It is clear from these findings that Hcy and vitamin B levels are related to MCI and dementia. However, to date, the relative contribution of each of the B vitamins and Hcy is not fully understood and deserves further research.

Extensive longitudinal studies on B vitamins and cognitive function have been conducted in Americans or Europeans, but limited research has examined the effects in the Chinese population. In a large American cohort entitled the Chicago Health and Aging Project (CHAP), which included 1041 residents aged 65 years or older who were followed for a median of 3.9 years for the development of AD [35], scholars did not find any association between quintile of B vitamin intake and risk of AD. The results were replicated in the Cache County Memory Study (CCMS) [36], another large cohort in the USA with 5092 men and women aged 65 years and older. The folate intake of these two American cohorts showed a similar median or mean intake level to ours, but the vitamin B_6_ and B_12_ intakes were much higher than ours. In a large French cohort [37], the Three-City Study, including 1321 older persons aged 75.8 years on average, higher folate intake was associated with a decreased risk of dementia, but no association was found for vitamin B_12_. It is noteworthy that compared with our cohort, the French cohort had a relatively low baseline folate status (average intake = 278 μg/day) but relatively adequate intakes of B_12_ (average intake = 5.7 μg/day). In a smaller British cohort with 155 participants aged 60–88 years [38], a lower dietary intake of vitamin B_6_ (0.9–1.4 mg/day) at baseline predicted a greater-than-expected rate of global cognitive decline (decrease in MMSE > 0.56 points per year). They concluded that vitamin B_6_ may be an important protective factor for maintaining cognitive health on the basis of a mean dietary intake of vitamin B_6_ (2.3 mg/day) similar to ours. Morris et al. [13] also observed that a higher intake of folate (median, 742 μg/day) than ours may have been associated with accelerated cognitive decline in 3718 Americans aged 65 years and older. Taken together, the inconsistencies between studies may be attributed to different intakes of B vitamins at baseline. As indicated by Smith and Refsum [15], the associations between the nutrient status and cognitive performance may follow a sigmoidal curve, which illustrates that additional nutrient intake is beneficial to a point but could be harmful at high intake, and it will have no effect at the plateau.

In our prospective study, the median intake of vitamin B_6_ was higher than the RNI, and folate intake was near the RNI, whereas intake of vitamin B_12_ was much less than the RNI. Consequently, significantly adverse effects on cognitive changes were only observed in vitamin B_12_ deficiency, whereas beneficial effects on cognitive reserve were observed in all three B vitamins and even harmful effects in the highest quartile of vitamin B_6_. However, in our secondary case-control analysis, MCI patients had significantly lower dietary intake of folate but equivalent intakes of vitamin B_6_ and B_12_ compared with controls, which was in line with their serum profiles and thus contributed to increased Hcy level in the MCI group. With respect to the serum level of Hcy, a systemic review and meta-analysis [39] revealed a positive trend between cognitive impairment and increased Hcy concentration. Meanwhile, the vast majority of case-control studies also observed significantly decreased blood folate levels in MCI and AD patients [40–44]. In contrast to folate, there are some discrepancies with regard to the blood level of vitamin B_12_. Despite the same trend for vitamin B_12_ as with folate in the above studies, many studies have found no significant differences between MCI/AD patients and healthy controls [32, 45–49], which was consistent with our results. The discrepant results indicate that the nutrient status of vitamin B_12_ in the subgroup of patients with cognitive impairment will not always be identical for the general population.

It is well documented that folate in one-carbon metabolism plays a central role in the synthesis, repair, and methylation of DNA, where it acts as a methyl donor. Folate homeostasis disruption could affect methylation potential through DNA gene hyper- or hypomethylation reactions and lead to gene transcription alterations (overexpression and/or gene silencing) [50, 51]. In our discovery group, genome-wide DNA methylation analysis identified that folate was significantly associated with DNA methylation of antioxidant genes NUDT15 and TXNRD1, both of which were hypermethylated in the MCI group. In addition to altered DNA methylation patterns, we found that MCI patients also had imbalanced redox status, as indicated by significantly increased levels of ROS, damage markers of DNA oxidation (8-OHdG), and lipid peroxidation (MDA and 8-iso-PGF2α), suggesting that the production of oxidant species overwhelmed the endogenous antioxidant ability to destroy them [52], which corresponded to the hypermethylation and low/no transcription or gene silencing of NUDT15 and TXNRD1.

NUDT15, also known as MTH2, is a member of the phosphatase protein family, which metabolizes a wide range of nucleotide substrates by hydrolyzing nucleoside triphosphates to their monophosphates and preventing the formation of 8-OHdG, the integration of the damaged purine nucleotides into DNA, and avoiding subsequent mismatch repair [53]. Lin et al. revealed that chronic hepatitis B virus X protein (HBx) could result in the accumulation of 8-OHdG in hepatocytes **by** inhibiting the expression of NUDT15 [54]. We also observed that the DNA methylation levels of CpG_19 located in TSS1500 (promoter region) in NUDT15 were positively correlated with ROS and 8-OHdG, indicating that the low/no transcription or gene silencing of NUDT15 was associated with increased DNA damage and decreased genome stability, which was implicated in the initiation and progression of neurodegenerative diseases [55]. The cytosolic selenoprotein thioredoxin reductase 1 (TrxR1, encoded by TXNRD1) is a member of the thioredoxin system that is indispensable for redox homoeostasis. With the support of several antioxidant systems, its physiologic functions may protect normal cells from oxidative stress [56]. We also found that the hypermethylated CpG_11 of TXNRD1 was positively correlated with 8-iso-PGF2α, an oxidative damage marker of lipid peroxidation in cell membranes, suggesting a dysregulation of TrxR1 owing to the downregulation of TXNRD1.

The methylation of CpG sites in NUDT15 and TXNRD1 was significantly correlated with folate and Hcy. Previous studies also investigated the relationship between folate and DNA methylation profile in redox-related genes in animal models and cells [57, 58]. They found that folate may suppress oxidative stress by inducing hypomethylation of BNIP3 and VPO1 but hypermethylation of EC-SOD, which probably indicated two important components of changes in DNA methylation. On the one hand, global hypomethylation, i.e., at most genes, demonstrated the role of folate as a methyl group donor and a reduction in the methylation of cytosine in DNA due to folate deficiency; on the other hand, focal hypermethylation might be affected by elevated Hcy levels. Such a phenomenon has been observed in carcinogenesis [59, 60].

The interaction of Hcy with NUDT15 and TXNRD1 was inversely associated with the cognitive performance of global cognition, processing speed, and attention. In contrast, the interaction between folate and TXNRD1 was positively associated with global cognition and immediate verbal memory. In line with our results, Fioravanti et al. [61] found significant improvement of memory and attention in folic acid supplementation among older participants selected for low initial folate in a small pilot trial. Durga et al. [62] conducted a randomized, double-blind, placebo-controlled study that included participants aged 50 years or above with high levels of Hcy and found significant beneficial effects of folic acid supplementation on memory and processing speed. Taken together, the results seem to demonstrate that disruption of the homeostasis of one-carbon metabolism induces altered DNA methylation patterns of NUDT15 and TXNRD1 and thus leads to oxidative stress overload and increased susceptibility to cognitive impairment.

Our findings indicate that the harmful role of vitamin B_12_ deficiency and beneficial effects of adequate folate intake in populations with relatively low basal vitamin B_12_ and folate status, such as middle-aged and elderly persons from China, a country with no B vitamin fortification and relatively low average intake levels of vitamin B_12_ and folate, may be worth exploring in future dementia prevention trials that implement food vitamin B_12_ and folate fortification programs to cover the requirements of the target population. Sanchez et al. [50] have reported that several countries, including Chile, started a policy of food folate and vitamin B_12_ fortification for older adults which led to elevated serum levels and decreased prevalence of deficiency. Cui et al. [57] demonstrated in vivo that folic acid supplementation may reduce oxidative stress and ROC levels by changing the DNA methylation of the oxidative stress-related gene VPO1. The validated genes in our study could also serve as potential novel targets to prevent dementia in MCI patients.

Given the interactive nature of nutrient metabolism and action and their different dietary sources, e.g., the vitamin B complex group, nutrient patterns (NPs) are advantageous since they capture the interactive effect of nutrients in combination [63]. Our previous cross-sectional study used exploratory factor analysis to generate three dietary nutrient intake combination patterns [9]. The first pattern was characterized as a “vitamin and mineral pattern” that included eight vitamins and six minerals, such as vitamin B_1_, vitamin B_2_, vitamin B_3_, vitamin B_6_, and folate. This pattern was identified as the most protective combination, with an adjusted odds ratio (OR) of 0.77 (0.71–0.83) for developing MCI. Our longitudinal studies with follow-ups will also generate and use NPs to explore and better define the cause-effect relationships between dietary nutrient intakes and cognitive decline as well as the distribution of benefits and harmful effects of the vitamin B complex group in this cohort.

A highlight of this study is the combination of prospective and case-control studies to investigate the effects of dietary and serum biomarker patterns of B vitamins in the general population and MCI patients, accompanied by mechanistic exploration. One limitation of the present study is the lack of RNA isolation to evaluate redox-related gene expression and its association with DNA methylation levels. However, information on oxidative damage markers suggests that hypermethylated redox-related genes were less expressed. The discovery group included a relatively smaller sample size, which may limit the candidate genes correlated with B vitamins. The lack of additional serum biomarkers of vitamin B_12_ (e.g., methylmalonic acid or holotranscobalamin) could have decreased the precision of status assessment. Finally, the method used to determine methylation status could have influenced the obtained results.

## Conclusion

Inadequate intake of vitamin B_12_ contributes to global and domain-specific cognitive decline, but adequate folate, vitamin B_6_, and vitamin B_12_ have beneficial effects on cognitive reserve in cognitively healthy persons. Decreased levels of circulating folate and increased Hcy were associated with hypermethylated redox-related genes and oxidative damage in MCI patients. The interaction between biomarker patterns of B vitamins and hypermethylated genes has significant effects on cognitive performance. These findings may provide unique leads for the combination of oxidative stress and DNA methylation when unraveling the mechanisms underlying the deleterious effects of B vitamin deficiency and hyperhomocysteinemia on cognition.

## Data Availability

The datasets during and/or analyzed during the current study are available from the corresponding author on reasonable request.

## Abbreviations

8-iso-PGF2α: 8-Iso-prostaglandin F2α
8-OHdG: 8-Hydroxy-desoxyguanosine
AD: Alzheimer’s disease
AVLT: Auditory Verbal Learning Test
BMI: Body mass index
CHD: Coronary heart disease
DSB: Digit span backward
DSF: Digit span forward
EMCOA: Effects and Mechanism Investigation of Cholesterol and Oxysterol on Alzheimer’s disease
FDR: False discovery rate
FFQ: Food Frequency Questionnaire
Hcy: Homocysteine
Hcy: Homocysteine
LMT: Logical Memory Test
MCI: Mild cognitive impairment
MDA: Malondialdehyde
MMSE: Mini-Mental State Examination
MoCA: Montreal Cognitive Assessment
NUDT15: Nudix hydrolase 15
RCT: Randomized controlled trials
ROS: Reactive oxygen species
SAM: *S*-adenosyl-methionine
SDMT: Symbol Digit Modalities Test
TXNRD1: Thioredoxin reductase 1
WMS-RC: Wechsler Memory Scale-Revised, Chinese version
